# Supplementary material for: Microbiota Composition of Breast Milk from Women of Different Ethnicity from the Manawatu—Wanganui Region of New Zealand
Source: Nutrients. 2020 Jun 11;12(6):1756. doi: 10.3390/nu12061756 (PMC7353441; doi:10.3390/nu12061756)
Supplement: Supplementary file 1 [file nutrients-12-01756-s001.pdf]

Supplementary material

Table S1. Demographics and baseline characteristics of the study participants.

Baseline characteristics	Mean	Range
Mothers (n = 78)		
Age (years)	31 ± 5	19–42
Weight (kg)	74 ± 14	48–109
Height (m)	1.65 ± 0.06	1.52–1.87
Body mass index (kg/m^2)	27 ± 5	20–39
Babies (n = 79)		
Birth weight (kg)	3.6 ± 0.5	2.4–4.6
Weight at sample collection (kg)	4.8 ± 0.6	3.3–6.2

Data expressed as mean ± standard deviation.

Table S2. Demographics and baseline characteristics of the study participants according to ethnicity.

	Asian	Māori & Pacific Island	New Zealand European	p value
Participants in group (n)	8	17	53	
Age (years)	30.4 ± 1.2	31.2 ± 1.5	30.7 ± 0.7	0.917
Weight (kg)	58.4 ± 3.1 ^{a}	80.8 ± 4.2 ^{b}	74.5 ± 1.6 ^{b}	<0.001
Height (m)	1.61 ± 0.02	1.65 ± 0.01	1.66 ± 0.01	0.162
Body mass index (kg/m^2)	22.5 ± 1.1 ^{a}	29.6 ± 1.5 ^{b}	27.2 ± 0.6 ^{b}	0.003
Birth weight (kg)	3.32 ± 0.13	3.63 ± 0.13	3.60 ± 0.06	0.255

Data expressed as mean ± standard error of the mean. Mean values with a different letter differ significantly, $p < 0.05$.

Table S3. World Health Organization and New Zealand Ministry of Health classifications of body mass index (kg/m^2).

	World Health Organisation [1]	New Zealand Ministry of Health [2]		
	All populations	Asian	Māori & Pacific Island	New Zealand European
Underweight	<18.50	<18.50	<18.50	<18.50
Normal	18.50-24.99	18.5-22.9	18.5-26	18.5-25
Overweight	≥25.00	23-27.4	26-32	25-30
Obese	≥30.00	>27.5	>32	>30

[1] Adapted from World Health Organization [1]. [2] Ministry of Health, New Zealand [2].

Table S4. Demographics and baseline characteristics of the study participants according to body mass index (BMI) classifications outlined by Ministry of Health, New Zealand.

	Normal	Overweight	Obese	p value
Age (years)	30.3 ± 1.0	31.5 ± 0.8	30.2 ± 1.4	0.559
Weight (kg)	60.4 ± 1.2 ^{a}	74.7 ± 1.7 ^{b}	92.0 ± 2.0 ^{c}	<0.001
Height (cm)	165.0 ± 1.4	165.2 ± 1.0	164.6 ± 1.1	0.943
Baby's weight (kg)	3.52 ± 0.08	3.65 ± 0.09	3.54 ± 0.14	0.592
Participants in BMI category (n)	27	31	20	

Data expressed as mean ± standard error of the mean. Mean values with a different letter differ significantly, $p < 0.05$.

Table S5. Relative abundance of bacteria at genus level in mother's breast milk categorized by body mass index.

	Normal	Overweight	Obese	Benjamini-Hochberg adjusted *p* value
Actinomyces	0.05 ± 0.04	0.76 ± 0.43	0.31 ± 0.12	0.760
Agrobacterium	0.71 ± 0.43	1.20 ± 1.09	0.06 ± 0.03	0.963
Akkermansia	1.96 ± 0.52	1.86 ± 0.39	1.63 ± 0.57	0.839
Anaerococcus	0.45 ± 0.27	0.10 ± 0.07	0.08 ± 0.05	0.760
Anaerostipes	0.06 ± 0.06	0	0.08 ± 0.08	0.964
Atopobium	0.01 ± 0.00	0.30 ± 0.19	0.74 ± 0.48	0.760
*Bacteroidales*_S24_7	0.01 ± 0.00	0.04 ± 0.02	0.10 ± 0.06	0.760
Bacteroides	3.07 ± 0.81	2.05 ± 0.51	3.44 ± 0.87	0.760
Barnesiellaceae	0.05 ± 0.05	0.04 ± 0.02	0.08 ± 0.05	0.760
Bifidobacteriaceae	0.09 ± 0.08	0.06 ± 0.03	0.17 ± 0.16	0.760
Bifidobacterium	6.94 ± 1.25	7.52 ± 0.98	7.63 ± 1.99	0.839
Blautia	2.60 ± 0.49	2.70 ± 0.38	2.39 ± 0.43	0.963
Brevundimonas	0.40 ± 0.40	0	0.13 ± 0.12	0.760
Caulobacteraceae	1.41 ± 0.56	1.31 ± 0.79	0.66 ± 0.55	0.760
Christensenella	0.08 ± 0.08	0	0	0.760
Christensenellaceae	0.42 ± 0.15	0.20 ± 0.06	0.23 ± 0.08	0.936
Chryseobacterium	2.03 ± 1.65	3.88 ± 2.13	0.11 ± 0.07	0.760
Clostridiaceae	1.01 ± 0.27	1.01 ± 0.22	0.85 ± 0.26	0.839
*Clostridiaceae*_Other	0.10 ± 0.06	0.19 ± 0.07	0.17 ± 0.09	0.760
Clostridiales	2.86 ± 0.59	3.40 ± 0.59	2.61 ± 0.45	0.839
*Clostridiales*_Other	0.46 ± 0.17	0.57 ± 0.13	0.52 ± 0.16	0.760
Clostridium	1.03 ± 0.42	1.47 ± 0.58	0.78 ± 0.38	0.760
Collinsella	1.30 ± 0.29	1.07 ± 0.18	1.13 ± 0.21	0.970
Coprococcus	1.58 ± 0.45	1.93 ± 0.32	1.91 ± 0.55	0.805
Coriobacteriaceae	0.36 ± 0.11	0.38 ± 0.12	0.29 ± 0.14	0.760
Corynebacterium	2.01 ± 0.63	2.21 ± 0.83	7.32 ± 3.20	0.760
Dorea	1.09 ± 0.50	0.59 ± 0.17	0.41 ± 0.21	0.760
Elizabethkingia	4.80 ± 3.32	0	0.056 ± 0.03	0.760
Enterobacteriaceae	0.05 ± 0.04	0.09 ± 0.07	0.42 ± 0.32	0.760
Epulopiscium	0	0.03 ± 0.03	0	0.760
Faecalibacterium	0.96 ± 0.21	1.21 ± 0.24	0.83 ± 0.22	0.848
Finegoldia	0.20 ± 0.13	0.10 ± 0.08	0.05 ± 0.03	0.760
Fusobacterium	0.51 ± 0.29	0.61 ± 0.31	0.50 ± 0.24	0.760
Lachnospira	1.04 ± 0.42	1.15 ± 0.26	0.62 ± 0.19	0.760
Lachnospiraceae	5.07 ± 1.18	6.38 ± 1.11	4.31 ± 0.96	0.839
*Lachnospiraceae*_Other	0.45 ± 0.22	0.35 ± 0.11	0.59 ± 0.16	0.760
Leucobacter	0.51 ± 0.45	0	0	0.760
Micrococcaceae	0.08 ± 0.08	0	0	0.760
Micrococcus	0.11 ± 0.10	0.03 ± 0.02	0	0.760
Mogibacteriaceae	0.18 ± 0.14	0.05 ± 0.03	0.01 ± 0.01	0.760
Odoribacter	0.07 ± 0.07	0	0	0.760
Oscillospira	0.75 ± 0.25	0.46 ± 0.13	0.56 ± 0.18	0.998
Parabacteroides	0.25 ± 0.10	0.29 ± 0.14	0.36 ± 0.11	0.760
Peptostreptococcus	0.40 ± 0.23	0.27 ± 0.12	0.22 ± 0.14	0.998
Porphyromonas	0.04 ± 0.03	0.21 ± 0.15	0.04 ± 0.04	0.760
Prevotella	0.03 ± 0.02	0.36 ± 0.22	0.19 ± 0.11	0.760
Propionibacterium	0.44 ± 0.17	1.58 ± 1.40	0.48 ± 0.19	0.760
Pseudomonas	0.99 ± 0.82	0.13 ± 0.08	4.56 ± 2.57	0.760
Rikenellaceae	2.72 ± 1.04	2.57 ± 1.02	4.17 ± 1.30	0.760
Roseburia	0.41 ± 0.13	0.63 ± 0.18	0.38 ± 0.17	0.760
Rothia	1.47 ± 0.59	1.84 ± 1.14	2.30 ± 1.11	0.760
Ruminococcaceae	11.4 ± 2.23	13.4 ± 2.09	9.73 ± 1.93	0.760
[*Ruminococcus*]	0.65 ± 0.24	1.49 ± 0.53	0.61 ± 0.22	0.760
Ruminococcus	1.56 ± 0.36	2.46 ± 0.49	1.40 ± 0.34	0.760

Sphingobium	1.21 ± 0.84	0.35 ± 0.11	0.16 ± 0.05	0.839
Sphingomonas	0.47 ± 0.18	0.28 ± 0.12	0.72 ± 0.34	0.760
Streptophyta	0.30 ± 0.16	0.22 ± 0.10	0.08 ± 0.07	0.760
Unassigned	1.53 ± 0.32	1.64 ± 0.23	4.80 ± 1.39	0.839
Veillonella	0.25 ± 0.12	0.19 ± 0.08	0.13 ± 0.07	0.982

Data expressed as mean ± standard error of the mean. Some taxonomy were unable to be identified at the genus level. Square brackets around a genus name indicate a candidate genus.

Table S6. Relative abundance of bacteria at genus level in mother's feces categorized according to the body mass index.

	**Normal**	**Overweight**	**Obese**	**Benjamini-Hochberg adjusted *p* value**
Actinomyces	0.022 ± 0.02	0	0	0.938
Agrobacterium	0	0.07 ± 0.07	0	0.938
Akkermansia	2.26 ± 0.91	3.74 ± 1.58	2.24 ± 0.83	0.938
Anaerococcus	0	0.05 ± 0.05	0	0.978
Anaerostipes	0.05 ± 0.02	0.09 ± 0.06	0.09 ± 0.03	0.938
Bacteroidales	0.39 ± 0.27	0	0	0.938
*Bacteroidales*_S24_7	1.13 ± 0.78	0.41 ± 0.31	0.15 ± 0.14	0.938
Bacteroides	17.7 ± 3.59	15.4 ± 2.92	20.5 ± 3.56	0.938
Barnesiellaceae	0.78 ± 0.25	1.04 ± 0.31	1.03 ± 0.30	0.938
Bifidobacterium	2.12 ± 0.55	1.80 ± 0.33	2.40 ± 1.20	0.938
Blautia	2.88 ± 0.74	2.96 ± 0.45	2.27 ± 0.32	0.938
Butyricimonas	0.14 ± 0.06	0.08 ± 0.03	0.16 ± 0.10	0.938
Caulobacteraceae	0.15 ± 0.10	0.21 ± 0.07	0.33 ± 0.18	0.967
Christensenellaceae	0.56 ± 0.14	0.68 ± 0.16	0.28 ± 0.09	0.938
Clostridiaceae	0.19 ± 0.05	0.42 ± 0.10	0.56 ± 0.19	0.938
*Clostridiaceae*_Other	0.03 ± 0.01	0.04 ± 0.01	0.07 ± 0.06	0.938
Clostridiales	5.28 ± 0.86	5.75 ± 0.72	3.35 ± 0.62	0.938
*Clostridiales*_Other	0.59 ± 0.13	1.24 ± 0.25	0.66 ± 0.10	0.938
Clostridium	0.30 ± 0.05	0.35 ± 0.05	0.31 ± 0.04	0.938
Collinsella	0.97 ± 0.34	0.76 ± 0.13	0.53 ± 0.12	0.938
Coprococcus	2.68 ± 0.41	3.27 ± 0.70	2.25 ± 0.30	0.938
Coriobacteriaceae	0.29 ± 0.08	0.42 ± 0.11	0.26 ± 0.07	0.938
Dorea	0.49 ± 0.10	1.04 ± 0.28	0.60 ± 0.09	0.938
Eggerthella	0.05 ± 0.02	0.03 ± 0.01	0.08 ± 0.05	0.938
Enterobacteriaceae	0.11 ± 0.07	0.07 ± 0.04	0	0.938
Epulopiscium	0.21 ± 0.21	0	0	0.938
Faecalibacterium	1.70 ± 0.43	1.42 ± 0.32	1.68 ± 0.41	0.938
Finegoldia	0.15 ± 0.05	0.19 ± 0.07	0.14 ± 0.06	0.938
Fusobacterium	0.01 ± 0.01	1.45 ± 1.43	0.01 ± 0.01	0.938
Lachnospira	1.89 ± 0.57	1.75 ± 0.61	1.31 ± 0.63	0.938
Lachnospiraceae	5.11 ± 0.96	6.65 ± 1.24	7.01 ± 1.25	0.938
*Lachnospiraceae*_Other	0.46 ± 0.09	0.52 ± 0.10	0.50 ± 0.11	0.978
Mogibacteriaceae	0.04 ± 0.02	0.08 ± 0.02	0.05 ± 0.03	0.938
Odoribacter	0.13 ± 0.03	0.16 ± 0.05	0.27 ± 0.09	0.938
Oscillospira	0.89 ± 0.20	0.94 ± 0.31	0.58 ± 0.12	0.941
Parabacteroides	1.92 ± 0.66	1.34 ± 0.36	1.61 ± 0.42	0.938
Paraprevotella	0.20 ± 0.16	0.13 ± 0.08	1.15 ± 0.98	0.938
Porphyromonas	0.04 ± 0.03	0.01 ± 0.01	0.01 ± 0.01	0.938
Prevotella	3.50 ± 2.03	0.50 ± 0.24	0.85 ± 0.55	0.938
Rikenellaceae	3.22 ± 0.94	3.03 ± 0.63	2.94 ± 0.72	0.967
*Rikenellaceae*_Other	0	0.03 ± 0.03	0	0.938
Roseburia	0.66 ± 0.53	0.59 ± 0.18	0.72 ± 0.33	0.938
Ruminococcaceae	22.2 ± 3.80	17.6 ± 3.08	25.9 ± 4.48	0.938
[*Ruminococcus*]	0.71 ± 0.18	1.78 ± 0.86	0.71 ± 0.18	0.967
Ruminococcus	0.29 ± 0.81	3.79 ± 0.95	3.80 ± 0.79	0.938
Sphingomonas	0.03 ± 0.02	0.01 ± 0.01	0.01 ± 0.01	0.938

Sutterella	0.12 ± 0.08	0.08 ± 0.07	0.02 ± 0.01	0.938
Unassigned	2.25 ± 0.25	2.23 ± 0.22	1.86 ± 0.16	0.938
Veillonella	1.26 ± 0.47	1.54 ± 0.50	2.40 ± 1.48	0.980

Data expressed as mean ± standard error of the mean. Some taxonomy were unable to be identified at the genus level. Square brackets around a genus name indicate a candidate genus.

Table S7. Relative abundance of bacteria at genus level in infant's feces categorized according to the mother's body mass index.

	**Normal**	**Overweight**	**Obese**	**Benjamini-Hochberg adjusted *p* value**
Actinomyces	1.31 ± 1.26	0.05 ± 0.03	1.67 ± 1.58	0.965
Agrobacterium	0	0.12 ± 0.08	0.01 ± 0.01	0.965
Akkermansia	0.12 ± 0.11	0.05 ± 0.03	0.01 ± 0.01	0.965
Bacteroides	19.9 ± 5.39	12.4 ± 4.03	24.8 ± 7.31	0.965
Bifidobacterium	45.5 ± 6.75	52.7 ± 6.29	32.8 ± 7.98	0.965
Blautia	0.65 ± 0.44	0.32 ± 0.17	0.08 ± 0.04	0.965
Caulobacteraceae	0.45 ± 0.45	2.59 ± 1.98	0.02 ± 0.02	0.965
Clostridiaceae	3.42 ± 2.75	3.34 ± 2.11	3.41 ± 3.25	0.965
*Clostridiaceae*_Other	0.03 ± 0.03	0.02 ± 0.01	0.01 ± 0.00	0.965
Clostridiales	0.78 ± 0.60	0.18 ± 0.09	0.13 ± 0.05	0.965
*Clostridiales*_Other	0.09 ± 0.06	0.04 ± 0.01	0.08 ± 0.04	0.965
Clostridium	5.79 ± 3.48	11.8 ± 5.23	17.6 ± 8.03	0.965
Collinsella	3.50 ± 1.93	1.50 ± 0.55	3.12 ± 1.76	0.965
Coprococcus	0.29 ± 0.18	0.21 ± 0.11	0.08 ± 0.04	0.965
Coriobacteriaceae	0.08 ± 0.05	0.79 ± 0.63	0.02 ± 0.01	0.965
Dorea	0.95 ± 0.93	0.23 ± 0.19	0.10 ± 0.08	0.988
Eggerthella	0.19 ± 0.10	0.05 ± 0.02	0.12 ± 0.07	0.965
Enterobacteriaceae	0.25 ± 0.08	0.40 ± 0.12	0.36 ± 0.11	0.965
Faecalibacterium	0.09 ± 0.06	0.06 ± 0.04	0.02 ± 0.01	0.965
Finegoldia	0.05 ± 0.05	0.02 ± 0.01	0.02 ± 0.02	0.965
Fusobacterium	0.65 ± 0.64	0.02 ± 0.01	0.01 ± 0.01	0.965
Lachnospiraceae	1.49 ± 0.59	0.25 ± 0.12	2.11 ± 1.41	0.965
*Lachnospiraceae*_Other	0.07 ± 0.02	0.03 ± 0.01	1.78 ± 1.72	0.965
Megasphaera	0	0.19 ± 0.14	0.10 ± 0.10	0.965
Odoribacter	0.09 ± 0.09	0.04 ± 0.04	0	0.965
Oscillospira	0.33 ± 0.19	0.08 ± 0.07	0.27 ± 0.25	0.965
Parabacteroides	1.71 ± 0.89	3.44 ± 2.29	0.99 ± 0.54	0.965
Peptostreptococcus	0.06 ± 0.05	0.01 ± 0.01	0.11 ± 0.11	0.965
Porphyromonas	0	0	0.15 ± 0.14	0.965
Prevotella	0.05 ± 0.02	0.02 ± 0.01	0.26 ± 0.22	0.965
Pseudoramibacter	0	0.16 ± 0.13	0	0.965
Rikenellaceae	0.59 ± 0.33	0.36 ± 0.24	0.04 ± 0.03	0.965
Ruminococcaceae	0.95 ± 0.44	2.27 ± 1.62	0.15 ± 0.04	0.979
[*Ruminococcus*]	3.89 ± 1.64	0.76 ± 0.36	5.35 ± 4.56	0.965
Ruminococcus	0.21 ± 0.13	0.18 ± 0.10	0.09 ± 0.03	0.965
Sphingomonas	0.50 ± 0.49	0.02 ± 0.02	0	0.965
Unassigned	2.70 ± 0.42	2.70 ± 0.48	2.43 ± 0.49	0.965
Veillonella	0.40 ± 0.26	0.40 ± 0.25	0.30 ± 0.18	0.979

Data expressed as mean ± standard error of the mean. Some taxonomy were unable to be identified at the genus level. Square brackets around a genus name indicate a candidate genus.

Table S8. Relative abundance of bacteria at genus level in mother's breast milk categorized according to the mode of delivery.

	Cesarean	Vaginal	Benjamini-Hochberg adjusted *p* value
Actinomyces	0.10 ± 0.05	0.46 ± 0.22	1.000
Agrobacterium	0.05 ± 0.04	0.88 ± 0.55	1.000
Akkermansia	1.77 ± 0.69	1.85 ± 0.30	1.000
Anaerococcus	0.03 ± 0.03	0.26 ± 0.12	1.000
Anaerostipes	0	0.05 ± 0.04	1.000
Atopobium	0.09 ± 0.07	0.36 ± 0.18	1.000
*Bacteroidales*_S24_7	0.03 ± 0.02	0.05 ± 0.02	1.000
Bacteroides	1.59 ± 0.57	3.03 ± 0.49	1.000
Barnesiellaceae	0	0.06 ± 0.03	1.000
Bifidobacteriaceae	0.19 ± 0.15	0.08 ± 0.05	1.000
Bifidobacterium	7.79 ± 2.87	7.25 ± 0.71	1.000
Blautia	2.00 ± 0.54	2.71 ± 0.28	1.000
Brevundimonas	0	0.21 ± 0.17	1.000
Caulobacteraceae	1.94 ± 1.53	1.01 ± 0.34	1.000
Christensenella	0	0.03 ± 0.03	1.000
Christensenellaceae	0.18 ± 0.10	0.31 ± 0.07	1.000
Chryseobacterium	3.39 ± 3.11	2.00 ± 1.04	1.000
Clostridiaceae	0.71 ± 0.27	1.03 ± 0.17	1.000
*Clostridiaceae*_Other	0.16 ± 0.11	0.15 ± 0.05	1.000
Clostridiales	2.78 ± 0.77	3.06 ± 0.37	1.000
*Clostridiales*_Other	0.43 ± 0.19	0.54 ± 0.10	1.000
Clostridium	0.67 ± 0.35	1.24 ± 0.34	1.000
Collinsella	1.17 ± 0.29	1.17 ± 0.15	1.000
Coprococcus	2.03 ± 0.59	1.75 ± 0.24	1.000
Coriobacteriaceae	0.15 ± 0.07	0.39 ± 0.08	1.000
Corynebacterium	4.15 ± 3.75	3.32 ± 0.82	1.000
Dorea	0.39 ± 0.15	0.79 ± 0.24	1.000
Elizabethkingia	0.04 ± 0.03	2.07 ± 1.44	1.000
Enterobacteriaceae	0.61 ± 0.46	0.06 ± 0.03	1.000
Epulopiscium	0	0.02 ± 0.01	1.000
Faecalibacterium	1.21 ± 0.44	0.98 ± 0.13	1.000
Finegoldia	0	0.15 ± 0.07	1.000
Fusobacterium	0.66 ± 0.41	0.52 ± 0.19	1.000
Lachnospira	0.89 ± 0.43	0.99 ± 0.21	1.000
Lachnospiraceae	5.28 ± 1.49	5.41 ± 0.72	1.000
*Lachnospiraceae*_Other	0.35 ± 0.16	0.47 ± 0.11	1.000
Leucobacter	0.86 ± 0.86	0.03 ± 0.03	1.000
Micrococcaceae	0	0.04 ± 0.04	1.000
Micrococcus	0.02 ± 0.02	0.06 ± 0.05	1.000
Mogibacteriaceae	0	0.10 ± 0.06	1.000
Odoribacter	0	0.03 ± 0.03	1.000
Oscillospira	0.68 ± 0.27	0.57 ± 0.12	1.000
Parabacteroides	0.19 ± 0.12	0.32 ± 0.08	1.000
Peptostreptococcus	0.03 ± 0.02	0.36 ± 0.12	1.000
Porphyromonas	0.06 ± 0.06	0.11 ± 0.07	1.000
Prevotella	0.13 ± 0.08	0.22 ± 0.11	1.000
Propionibacterium	0.12 ± 0.05	1.06 ± 0.67	1.000
Pseudomonas	1.18 ± 1.01	1.68 ± 0.88	1.000
Rikenellaceae	2.28 ± 1.12	3.20 ± 0.73	1.000
Roseburia	0.40 ± 0.20	0.51 ± 0.11	1.000
Rothia	0.46 ± 0.17	2.13 ± 0.68	1.000
Ruminococcaceae	13.0 ± 3.48	11.5 ± 1.31	1.000
[*Ruminococcus*]	1.68 ± 1.06	0.81 ± 0.17	1.000
Ruminococcus	2.32 ± 0.87	1.77 ± 0.24	1.000

Sphingobium	0.77 ± 0.51	0.57 ± 0.35	1.000
Sphingomonas	0.64 ± 0.36	0.42 ± 0.12	1.000
Streptophyta	0.05 ± 0.05	0.24 ± 0.08	1.000
Unassigned	2.99 ± 1.40	2.30 ± 0.41	1.000
Veillonella	0.23 ± 0.19	0.20 ± 0.05	1.000

Data expressed as mean ± standard error of the mean. Some taxonomy were unable to be identified at the genus level. Square brackets around a genus name indicate a candidate genus.

Table S9. Relative abundance of bacteria at genus level in mother's feces categorized according to the mode of delivery.

	**Cesarean**	**Vaginal**	**Benjamini-Hochberg adjusted *p* value**
Actinomyces	0	0.01 ± 0.01	0.935
Agrobacterium	0	0.04 ± 0.04	0.935
Akkermansia	2.86 ± 1.23	2.83 ± 0.84	0.935
Anaerococcus	0	0.03 ± 0.03	0.935
Anaerostipes	0.19 ± 0.13	0.05 ± 0.01	0.935
Bacteroidales	0	0.17 ± 0.12	0.964
*Bacteroidales*_S24_7	0.85 ± 0.66	0.54 ± 0.34	0.935
Bacteroides	16.8 ± 3.70	17.7 ± 2.21	0.935
Barnesiellaceae	0.73 ± 0.25	0.99 ± 0.20	0.964
Bifidobacterium	1.60 ± 0.57	2.17 ± 0.45	0.935
Blautia	3.34 ± 0.92	2.63 ± 0.34	0.935
Butyricimonas	0.04 ± 0.02	0.14 ± 0.04	0.935
Caulobacteraceae	0.37 ± 0.24	0.19 ± 0.06	0.935
Christensenellaceae	0.38 ± 0.16	0.56 ± 0.10	0.935
Clostridiaceae	0.62 ± 0.22	0.32 ± 0.06	0.935
*Clostridiaceae*_Other	0.03 ± 0.02	0.05 ± 0.02	0.935
Clostridiales	5.03 ± 1.09	4.95 ± 0.50	0.973
*Clostridiales*_Other	1.29 ± 0.47	0.76 ± 0.10	0.935
Clostridium	0.43 ± 0.08	0.29 ± 0.03	0.935
Collinsella	0.52 ± 0.11	0.83 ± 0.16	0.935
Coprococcus	2.73 ± 0.45	2.82 ± 0.38	0.935
Coriobacteriaceae	0.41 ± 0.11	0.33 ± 0.06	0.935
Dorea	0.79 ± 0.19	0.72 ± 0.14	0.935
Eggerthella	0.03 ± 0.01	0.06 ± 0.02	0.964
Enterobacteriaceae	0.01 ± 0.00	0.08 ± 0.04	0.935
Epulopiscium	0	0.09 ± 0.09	0.935
Faecalibacterium	1.93 ± 0.57	1.51 ± 0.24	0.935
Finegoldia	0.12 ± 0.07	0.17 ± 0.04	0.935
Fusobacterium	0	0.70 ± 0.68	0.935
Lachnospira	1.77 ± 0.84	1.67 ± 0.38	0.935
Lachnospiraceae	9.68 ± 2.14	5.43 ± 0.64	0.935
*Lachnospiraceae*_Other	0.70 ± 0.17	0.45 ± 0.06	0.935
Mogibacteriaceae	0.04 ± 0.02	0.06 ± 0.02	0.935
Odoribacter	0.17 ± 0.05	0.18 ± 0.04	0.964
Oscillospira	0.65 ± 0.14	0.87 ± 0.17	0.964
Parabacteroides	1.05 ± 0.32	1.74 ± 0.35	0.935
Paraprevotella	0.23 ± 0.17	0.46 ± 0.32	0.935
Porphyromonas	0.01 ± 0.01	0.03 ± 0.01	0.935
Prevotella	1.13 ± 0.79	1.76 ± 0.89	0.997
Rikenellaceae	3.78 ± 0.96	2.92 ± 0.50	0.935
*Rikenellaceae*_Other	0	0.02 ± 0.01	0.964
Roseburia	0.53 ± 0.27	0.68 ± 0.26	0.935
Ruminococcaceae	18.0 ± 3.75	22.1 ± 2.48	0.935
[*Ruminococcus*]	1.22 ± 0.52	1.11 ± 0.41	0.935
Ruminococcus	5.61 ± 1.51	3.01 ± 0.51	0.935
Sphingomonas	0.02 ± 0.01	0.02 ± 0.01	0.935

Sutterella	0.17 ± 0.14	0.06 ± 0.03	0.935
Unassigned	2.19 ± 0.26	2.13 ± 0.15	0.935
Veillonella	2.76 ± 1.95	1.42 ± 0.36	0.964

Data expressed as mean ± standard error of the mean. Some taxonomy were unable to be identified at the genus level. Square brackets around a genus name indicate a candidate genus.

Table S10. Relative abundance of bacteria in infant's feces at genus level categorized according to the mode of delivery.

	Cesarean	Vaginal	Benjamini-Hochberg adjusted *p* value
Actinomyces	2.74 ± 2.61	0.52 ± 0.48	0.848
Agrobacterium	0.13 ± 0.12	0.03 ± 0.03	0.557
Akkermansia	0.08 ± 0.07	0.06 ± 0.05	0.848
Anaerotruncus	0	0.02 ± 0.02	0.848
Bacteroides	6.28 ± 5.38	20.6 ± 3.51	0.104
Bifidobacterium	41.7 ± 10.8	45.9 ± 4.36	0.848
Blautia	0.98 ± 0.88	0.25 ± 0.10	0.850
Caulobacteraceae	0.15 ± 0.15	1.40 ± 0.96	0.614
Clostridiaceae	6.13 ± 4.77	2.82 ± 1.53	0.104
*Clostridiaceae*_Other	0.01 ± 0.01	0.02 ± 0.01	0.848
Clostridiales	0.18 ± 0.10	0.42 ± 0.26	0.947
*Clostridiales*_Other	0.04 ± 0.02	0.06 ± 0.03	0.848
Clostridium	18.6 ± 9.36	9.55 ± 3.26	0.557
Collinsella	0.02 ± 0.02*	3.15 ± 1.00*	<0.001
Coprococcus	0.09 ± 0.06	0.23 ± 0.09	0.850
Coriobacteriaceae	0.01 ± 0.01	0.42 ± 0.30	0.532
Dorea	0.52 ± 0.43	0.44 ± 0.40	0.782
Eggerthella	0.03 ± 0.02	0.14 ± 0.05	0.532
Enterobacteriaceae	0.52 ± 0.16	0.30 ± 0.07	0.484
Faecalibacterium	0.11 ± 0.08	0.05 ± 0.03	0.848
Finegoldia	0	0.03 ± 0.02	0.490
Fusobacterium	1.36 ± 1.32	0.01 ± 0.01	0.312
Lachnospiraceae	1.41 ± 0.84	1.10 ± 0.41	0.782
*Lachnospiraceae*_Other	0.10 ± 0.04	0.56 ± 0.52	0.490
Megasphaera	0	0.12 ± 0.07	0.848
Odoribacter	0	0.06 ± 0.04	0.615
Oscillospira	0.01 ± 0.01	0.26 ± 0.12	0.848
Parabacteroides	5.16 ± 5.15	1.61 ± 0.51	0.615
Peptostreptococcus	0	0.06 ± 0.04	0.848
Porphyromonas	0	0.05 ± 0.04	0.557
Prevotella	0.01 ± 0.00	0.11 ± 0.07	0.848
Pseudoramibacter	0	0.08 ± 0.06	0.848
Rikenellaceae	0.01 ± 0.00	0.43 ± 0.18	0.850
Ruminococcaceae	0.73 ± 0.55	1.38 ± 0.79	0.848
[*Ruminococcus*]	7.88 ± 6.63	2.02 ± 0.75	0.507
Ruminococcus	0.28 ± 0.22	0.14 ± 0.06	0.848
Sphingomonas	0	0.22 ± 0.21	0.848
Unassigned	3.05 ± 0.97	2.55 ± 0.26	0.848
Veillonella	0.69 ± 0.34	0.31 ± 0.15	0.532

Data expressed as mean ± standard error of the mean. Some taxonomy were unable to be identified at the genus level. Square brackets around a genus name indicate a candidate genus. Mean values marked with * differ significantly, $p < 0.05$.

Table S11. Summary of immune modulatory proteins analyzed, their potential impact on infant health, and maternal diet and lifestyle influences affecting their concentrations in breast milk.

Breast milk proteins analyzed	Potential role	Reference
Interleukin 2	Role in T cell differentiation	[3]
	Diet can impact the amount present in breast milk	[4]
Interleukin 4	Role in allergies and immunoglobulin (Ig) E production	[5]
	Higher in milk when maternal allergies are present	[6]
	Higher in mature milk of smokers	[7]
Interleukin 6	Predominant cytokine in milk	[7]
	Has a role in the differentiation of IgA producing cells	[8]
	Higher in mature milk of smokers	[7]
	Lower in milk when maternal allergies are present	[7]
Interleukin 7	Regulatory cytokine that can cross the intestinal wall and influence thymic development in infants	[9]
Interleukin 8	Role in neutrophil recruitment	[10]
	High levels indicate subclinical breast inflammation leading to poor infant growth	[11]
	High amounts in mature milk of mothers who had pre-eclampsia	[12]
	Decreased in milk when maternal rhinitis is present	
Interleukin 10	Plays a role in oral tolerance	
	Involved in IgA synthesis	
Interferon γ	Has a critical role in innate and adaptive immunity	[13]
	Recruits macrophages	[13]
	Higher in mature milk of smokers	[7]
	Increases as milk matures	[4]
	Remains low in infants that develop atopy	[14]
Insulin-like growth factor 1	High amounts in milk associated with high weight gain in infants	[15]
Transforming growth factor β1	Promotes functional development of the gastrointestinal mucosa	[16]
	Plays a role in oral tolerance	[17]
Transforming growth factor β2	Predominant cytokine in milk	[6]
	Lower in milk when maternal allergies are present	[18]
	Lower in milk with increased maternal body mass index (BMI), but not significant	[19]
	Milk concentrations correlate with maternal polyunsaturated fatty acid consumption	[18]
Soluble cluster of differentiation 14	Enhances cellular response to microbial components	[20]
	Lower amounts in human milk as it matures	[19]
	Higher amounts in milk are associated with bifidobacteria and staphylococci	[19]
	Low amounts in milk is associated with high BMI	[19]
Immunoglobulin A	Binds microbial antigens	[8,19]
	Enhances phagocytosis	[8]
	Promotes normal microbial colonization in the gut	[8]

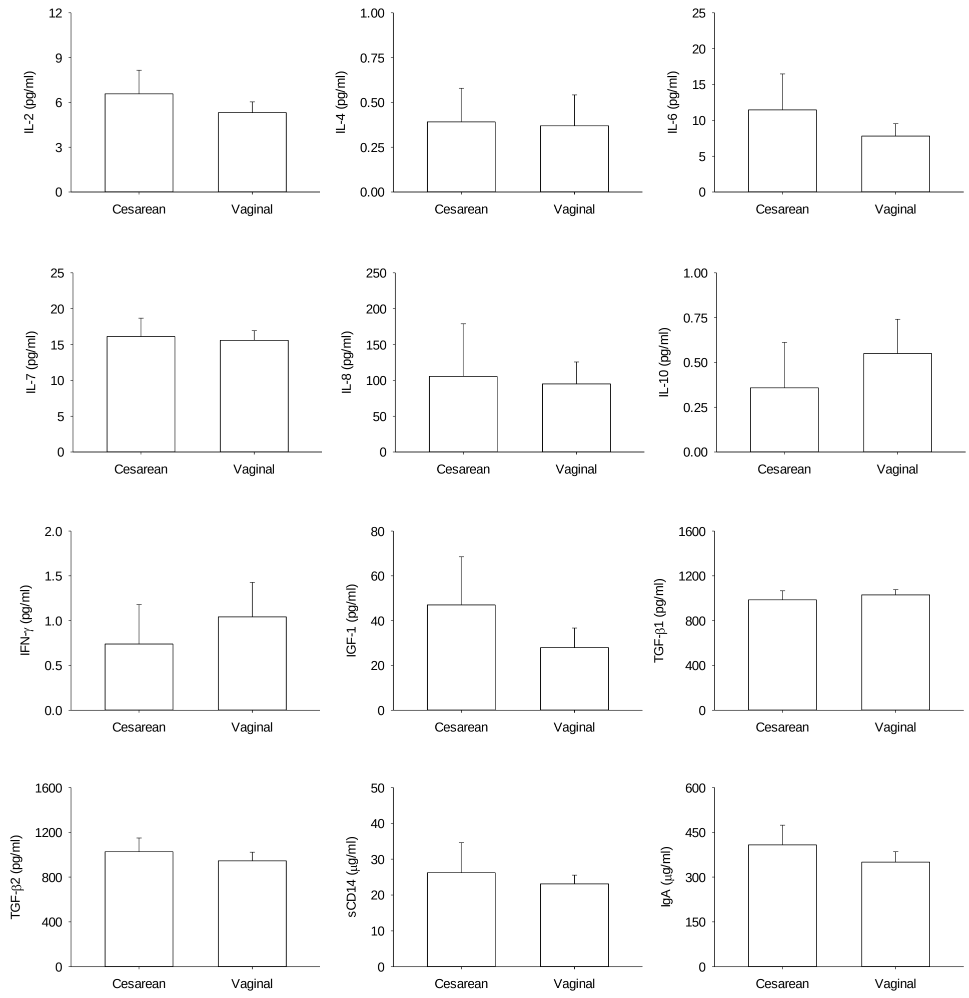

Figure S1. Immune related proteins in mother's breast milk categorized according to the mode of delivery of infants. Data expressed as mean ± standard error of the mean. IL – Interleukin, IFN – Interferon, IGF – Insulin-like growth factor, TGF – Transforming growth factor, sCD – Soluble cluster of differentiation, Ig – Immunoglobulin.

References

1. World Health Organization. BMI Classification. In *Global Database on Body Mass Index*, 2006 ed.; World Health Organisation: Geneva, 2006.
2. Ministry of Health. Food and Nutrition Guidelines for Healthy Pregnant and Breastfeeding Women: A background paper. Ministry of Health: Wellington, 2006.
3. Ross, S.H.; Cantrell, D.A. Signaling and Function of Interleukin-2 in T Lymphocytes. *Annual Review of Immunology* **2018**, *36*, 411-433.
4. Hoppu, U.; Isolauri, E.; Laakso, P.; Matomaki, J.; Laitinen, K. Probiotics and dietary counselling targeting maternal dietary fat intake modifies breast milk fatty acids and cytokines. *European Journal of Nutrition* **2012**, *51*, 211-219.
5. Steinke, J.W.; Borish, L. Th2 cytokines and asthma. Interleukin-4: its role in the pathogenesis of asthma, and targeting it for asthma treatment with interleukin-4 receptor antagonists. *Respir Res* **2001**, *2*, 66-70.
6. Bottcher, M.F.; Jenmalm, M.C.; Garofalo, R.P.; Bjorksten, B. Cytokines in breast milk from allergic and nonallergic mothers. *Pediatr. Res.* **2000**, *47*, 157-162.
7. Burch, J.L.; Soto-Ramirez, N.; Karmaus, W. Breast Milk Cytokines and Maternal Allergy. *J. Allergy Clin. Immunol.* **2011**, *127*, AB129.

8. Castellote, C.; Casillas, R.; Ramirez-Santana, C.; Perez-Cano, F.J.; Castell, M.; Gloria Moretones, M.; Lopez-Sabater, M.C.; Franch, A. Premature Delivery Influences the Immunological Composition of Colostrum and Transitional and Mature Human Milk. *J. Nutr.* **2011**, *141*, 1181-1187.
9. Ngom, P.T.; Collinson, A.C.; Pido-Lopez, J.; Henson, S.M.; Prentice, A.M.; Aspinall, R. Improved thymic function in exclusively breastfed infants is associated with higher interleukin 7 concentrations in their mothers' breast milk. *Am. J. Clin. Nutr.* **2004**, *80*, 722-728.
10. Meki, A.-R.M.A.; Saleem, T.H.; Al-Ghazali, M.H.; Sayed, A.A. Interleukins -6, -8 and -10 and tumor necrosis factor-alpha and its soluble receptor I in human milk at different periods of lactation. *Nutr. Res.* **2003**, *23*, 845-855.
11. Filteau, S.M.; Lietz, G.; Mulokozi, G.; Bilotta, S.; Henry, C.J.K.; Tomkins, A.M. Milk cytokines and subclinical breast inflammation in Tanzanian women: effects of dietary red palm oil or sunflower oil supplementation. *Immunology* **1999**, *97*, 595-600.
12. Erbağcı, A.B.; Çekmen, M.B.; Balat, Ö.; Balat, A.; Aksoy, F.; Tarakçıoğlu, M. Persistency of high proinflammatory cytokine levels from colostrum to mature milk in preeclampsia. *Clin. Biochem.* **2005**, *38*, 712-716.
13. Teixeira, L.K.; Fonseca, B.P.; Barboza, B.A.; Viola, J.P. The role of interferon-g on immune and allergic responses. *Memórias do Instituto Oswaldo Cruz* **2005**, *100*, 137-144.
14. Tang, M.L.K.; Kemp, A.S.; Hill, D.J.; Thorburn, J. Reduced interferon-γ secretion in neonates and subsequent atopy. *The Lancet* **1994**, *344*, 983-985.
15. Kon, I.Y.; Shilina, N.M.; Gmoshinskaya, M.V.; Ivanushkina, T.A. The Study of Breast Milk IGF-1, Leptin, Ghrelin and Adiponectin Levels as Possible Reasons of High Weight Gain in Breast-Fed Infants. *Ann. Nutr. Metab.* **2014**, *65*, 317-323.
16. Alasmar, R.M.; Varadharajan, K.; Shanmugakonar, M.; Al-Naemi, H.A. Gut Microbiota and Health: Understanding the Role of Diet. *Food and Nutrition Sciences* **2019**, *Vol.10No.11*, 29.
17. Penttila, I. Effects of transforming growth factor-beta and formula feeding on systemic immune responses to dietary beta-lactoglobulin in allergy-prone rats. *Pediatr. Res.* **2006**, *59*, 650-655.
18. Laiho, K.; Lampi, A.M.; Hamalainen, M.; Moilanen, E.; Piironen, V.; Arvola, T.; Syrjanen, S.; Isolauri, E. Breast milk fatty acids, eicosanoids, and cytokines in mothers with and without allergic disease. *Pediatr. Res.* **2003**, *53*, 642-647.
19. Collado, M.C.; Laitinen, K.; Salminen, S.; Isolauri, E. Maternal weight and excessive weight gain during pregnancy modify the immunomodulatory potential of breast milk. *Pediatric Research* **2012**, *72*, 77-85.
20. Snijders, B.E.P.; Damoiseaux, J.; Penders, J.; Kummeling, I.; Stelma, F.F.; van Ree, R.; van den Brandt, P.A.; Thijs, C. Cytokines and soluble CD14 in breast milk in relation with atopic manifestations in mother and infant (KOALA Study). *Clin. Exp. Allergy* **2006**, *36*, 1609-1615.
